# Thermal conductance between water and nm-thick WS_2_: extremely localized probing using nanosecond energy transport state-resolved Raman†

**DOI:** 10.1039/d0na00844c

**Published:** 2020-11-02

**Authors:** Hamidreza Zobeiri, Nicholas Hunter, Ridong Wang, Xinman Liu, Hong Tan, Shen Xu, Xinwei Wang

**Affiliations:** Department of Mechanical Engineering, Iowa State University Ames Iowa 50011 USA shxu16@sues.edu.cn xwang3@iastate.edu +1-515-294-8023; State Key Laboratory of Precision Measuring Technology and Instruments, Tianjin University Tianjin 300072 P. R. China; Department of Landscape Architecture, University of Washington Seattle Washington 98105 USA; School of Energy and Power Engineering, Nanjing University of Science and Technology Nanjing 210094 P. R. China tanhongwh@njust.edu.cn; Automotive Engineering College, Shanghai University of Engineering Science 333 Longteng Road Shanghai 201620 People's Republic of China

## Abstract

Liquid–solid interface energy transport has been a long-term research topic. Past research mostly focused on theoretical studies while there are only a handful of experimental reports because of the extreme challenges faced in measuring such interfaces. Here, by constructing nanosecond energy transport state-resolved Raman spectroscopy (nET-Raman), we characterize thermal conductance across a liquid–solid interface: water–WS_2_ nm film. In the studied system, one side of a nm-thick WS_2_ film is in contact with water and the other side is isolated. WS_2_ samples are irradiated with 532 nm wavelength lasers and their temperature evolution is monitored by tracking the Raman shift variation in the E_2g_ mode at several laser powers. Steady and transient heating states are created using continuous wave and nanosecond pulsed lasers, respectively. We find that the thermal conductance between water and WS_2_ is in the range of 2.5–11.8 MW m^−2^ K^−1^ for three measured samples (22, 33, and 88 nm thick). This is in agreement with molecular dynamics simulation results and previous experimental work. The slight differences are attributed mostly to the solid–liquid interaction at the boundary and the surface energies of different solid materials. Our detailed analysis confirms that nET-Raman is very robust in characterizing such interface thermal conductance. It completely eliminates the need for laser power absorption and Raman temperature coefficients, and is insensitive to the large uncertainties in 2D material properties input.

## Abstract 2

Interfacial thermal conductance between a nm-thick suspended WS_2_ film and water is measured using a novel nET-Raman technique. By significantly reducing the effect of water thermal resistance, the interface resistance effect become more preeminent.

## Graphical abstract

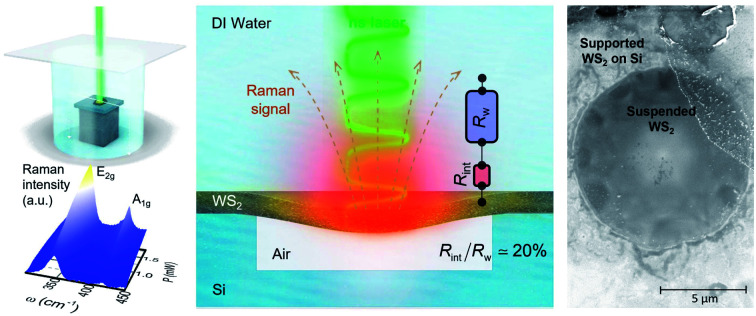

## 1. Introduction

Thermal transport across a solid–liquid interface is a topic of ongoing research due to its various applications in micro/nanoscale thermal transport, such as evaporation cooling and energy conversion,^1–5^ thermal management,^6–8^ ultrafast flow delivery,^9^ cancer treatment,^10^ solar thermal heating,^11^ and nanofluids.^12,13^

Continuum based interface thermal resistance (ITR) models describe this resistance as an irruption on phonon propagation in a crystalline lattice. This is due to the difference in the speed of sound between two materials which leads to a mismatch in acoustic impedance.^14^ The Acoustic Mismatch Model (AMM) and the Diffuse Mismatch Model (DMM) are the main models to explain this mismatch across a solid–liquid interface and have been used widely for the theoretical calculation of interface thermal transport.^15^ The AMM model neglects phonon scattering at the interface, while the DMM model considers their diffuse scattering across the interface.^16,17^ AMM and DMM predict high and low interface thermal resistance, respectively, which provide upper and lower limits for the interface thermal resistance. However, these two models do not consider surface complexities and solid–liquid interaction strength. Molecular dynamic (MD) simulation is an alternative method for studying ITR theoretically without considering continuum based governing equations, and it is capable of studying several factors that can affect the ITR, such as surface wettability. Note that in some calculations the term Kapitza length *l*_K_ is used to represent the ITR quantitatively. *l*_K_ is defined as: *l*_*K*_ = *R*_K_*k*, where *R*_K_ is ITR or Kapitza resistance and *k* is the thermal conductivity of one of the phases, usually the liquid. Barrat *et al.* studied the dependence of *R*_K_ on wetting properties using non-equilibrium MD simulation as a function of the interaction coefficient (*c*_12_) of the Lennard-Jones equation and under normal pressures. Their results showed relatively large values of *R*_K_ when the liquid is not wetting the solid (small *c*_12_ values).^18^ They reported that *l*_K_ decreased from 50 nm to less than 10 nm, as the *c*_12_ coefficient increased from 0.5 to 1. Kim *et al.* investigated the interface thermal transport between parallel plates separated by a thin layer of liquid argon using a 3D MD simulation employing 6–12 Lennard-Jones potential interactions, and studied *l*_K_ as a function of surface wettability, thermal oscillation frequency, wall temperature (from 80 to 160 K), and channel height. They assumed that the solid molecules had the same mass as the argon molecules. Their results indicated that *l*_K_ varies from 1 to 10 nm under several scenarios.^19^ Similar results were reported by Giri *et al.* and Vo *et al.* regarding the effect of interaction strength and thermal boundary conductance.^20,21^ In another work, *R*_K_ was reported in the range of 5 × 10^−8^ to 4 × 10^−7^ m^2^ K W^−1^ using non-equilibrium MD simulations at liquid–vapor Ar mixtures adjacent to warmer Fe walls.^22^ Murad *et al.* studied the ITR between Si and water using MD simulation, and they found that *R*_K_ decreases with increasing temperature from 5 × 10^−6^ m^2^ K W^−1^ to 3 × 10^−9^ m^2^ K W^−1^ when temperature increases from ∼350 K to ∼550 K.^23^ In the work by Shenogina *et al.*, it is reported that the Kapitza conductance is proportional to the work of adhesion, and for a highly hydrophilic surface it can be up to ∼160 MW m^−2^ K^−1^.^24^ Barisik *et al.* performed MD simulations of heat conduction in liquid Ar that is confined in Ag nano-channels and reported that *R*_K_ can vary from 0.8 × 10^−9^ to 5 × 10^−9^ m^2^ K W^−1^ from cold to hot surface temperature, respectively.^25^ In another work they utilized MD simulations to study ITR at Ar–Ag and Ar–graphite interfaces, and concluded that *l*_K_ increases with increased wall temperature, and is three times larger at an Ar–graphite interface than that at an Ar–Ag interface which is due to the difference between the interaction potentials of the molecular pairs in the two cases.^16^ While the last two works were conducted under generally low temperatures (∼130 K), Barisik *et al.* conducted other MD simulations and reported that *l*_K_ at Si–water in a higher temperature range (more than RT) decreases slightly with increased wall temperature, and is on average around 9 nm.^26^ The pressure dependence of ITR at Au–water and Si–water interfaces was studied using MD simulations by Pham *et al.*^27^ Their results revealed that the pressure dependence of *l*_K_ depends on surface wettability. The *l*_K_ of the Au–water (hydrophobic) interface was stable despite increasing water pressure, while it changed significantly across an Si–water interface (hydrophilic). Han *et al.* drew the same conclusion that ITR increases with liquid pressure enhancement through an MD simulation of *n*-perfluorohexane in contact with gold.^28^ The ITRs of several linear alkane liquids in contact with gold were obtained using non-equilibrium MD by Bin Saleman *et al.* They found that ITR is directly proportional to the number of carbon atoms in an alkane molecule and on average is ∼1.5 × 10^−7^ m^2^ K W^−1^.^29^

Past discussion was mostly focused on theoretical works, especially MD simulations. Unfortunately, there are only a few experimental works in the field of solid–liquid ITR measurement to compare with those calculated values. In 2002, M. Wilson *et al.* investigated thermal interface conductance between Au, Pt, and AuPd nanoparticles suspended in water or toluene. They found a thermal conductance (*G*) of 130 MW m^−2^ K^−1^ for a citrate-stabilized Pt nanoparticles and water interface by heating particles with a 770 nm optical laser and interrogating the decay of their temperature through time-resolved changes in optical absorption.^30^ In their next work, the effect of the organic stabilizing group on the *G* of AuPd particle–water and AuPd particle–toluene interfaces was studied with a similar technique.^31^ Two conclusions were arrived at in their work: (1) the values of *G* of the particle–water interface under different stabilizing groups were in the order of 100–300 MW m^−2^ K^−1^, which means that *G* is large, regardless of the self-assembled stabilizing group, and (2) the *G* of an AuPd particle–water interface was larger than that of an AuPd particle–toluene interface, which indicates the effect of the liquid phase on ITR. In another work, Ge *et al.* performed a similar time-domain thermoreflectance technique and studied the effects of surface wettability on *l*_K_ using Au and Al based surfaces. The results indicated that *l*_K_ at hydrophobic (Al) interfaces (10–12 nm) is a factor of 2–3 larger than *l*_K_ at hydrophilic (Au) interfaces (3–6 nm), which is in agreement with MD simulations.^32^ Park *et al.* reported ITR studies for a system of Au nanorods immobilized on a crystalline quartz support and immersed in various organic fluids by heating the nanorods with a subpicosecond optical pulse and monitoring their cooling process by transient absorption.^33^ They found the thermal conductances of the nanorod–fluid interface at 36 ± 4 MW m^−2^ K^−1^, 32 ± 6 MW m^−2^ K^−1^, 30 ± 5 MW m^−2^ K^−1^, and 35 ± 4 MW m^−2^ K^−1^, for methanol, ethanol, toluene, and hexane, respectively. This indicated that *G* drops significantly as water is replaced by an organic fluid. Using a similar technique, it was reported that the *G* of Au nanodisks coated with a hydrophilic self-assembled monolayer varies over 90–190 MW m^−2^ K^−1^, depending on the amount of water in the liquid mixture. For hydrophobic surfaces, *G* is in range of 70 ± 10 MW m^−2^ K^−1^. This was attributed to the effects of the work of adhesion on interface thermal conductance.^34^

Raman spectroscopy has proved to be a powerful tool for studying thermal transport at micro/nanoscales. Several works have been reported that show the potential of this tool to investigate the thermal conductivity and hot carrier diffusion coefficient of 2D materials, such as graphene^35,36^ and transition metal dichalcogenides (TMD).^37–40^ Raman spectroscopy is able to measure the ITR of solid–solid interfaces, as well as the aforementioned properties. Yuan *et al.* reported the interface thermal conductance between few-layered to multi-layered MoS_2_ films and Si, and showed that *G* increases with an increased number of layers of MoS_2_ thin film from 1 to 69 MW m^−2^ K^−1^.^41^ They reported other works that successfully measured the ITR between thin layers of TMD materials and a glass or Si substrate.^42–44^ Raman spectroscopy based techniques have the advantage of being non-contact, non-invasive, and material-specific leading to higher accuracy of measured parameters.

In this work, for the first time, the interfacial thermal conductance (*G*_int_) between de-ionized (DI) water and WS_2_ nm-thick film is measured using a novel nanosecond energy transport resolved Raman (nET-Raman) technique. Each WS_2_ sample is suspended over a hole, and immersed in a water bath. Using this experimental structure, WS_2_ film is in contact with water from the top, while its other side is isolated thermally by air inside the hole. Interfacial thermal transport between solid and liquid is characterized here for three samples of different thicknesses. The measured *G*_int_ is compared and verified with other literature values based on both experimental and MD methods. It is shown in detail that the accuracy of the measurement can be improved by using shorter laser pulses as the transient part of the Raman thermometry. Also, it is proved that uncertainties in the laser absorption coefficient, Raman temperature coefficient, and values of thermal properties of WS_2_ film in theoretical calculations do not downgrade the precision of characterization. In the following, the feasibility and capability of this method are explored in detail.

## 2. Materials preparation and theoretical basis

### 2.1. Sample preparation

**Fig. 1:**
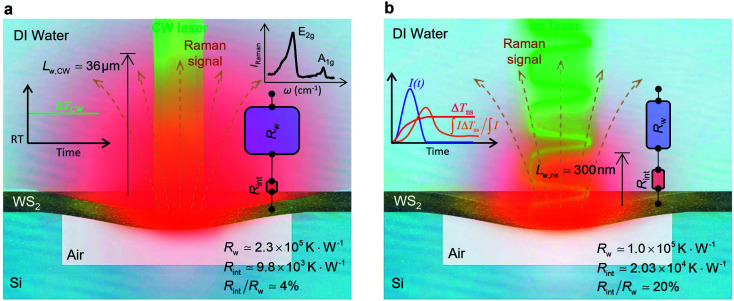
Cross-sectional view of the experimental sample design to measure the interfacial thermal conductance (*G*_int_) at a water–WS_2_ nm-thick film interface. The nm-thick WS_2_ film is suspended over a hole in an Si layer. The hole depth is 3 μm. A graphical illustration of the effects of relative contribution of total interface resistance (*R*_int_) and water thermal resistance (*R*_w_) under (a) CW and (b) ns heating states. Under each state, the WS_2_ film is irradiated using a specific laser and the Raman signal is collected. A sample Raman spectrum of WS_2_ is shown in the inset of figure (a). Under CW laser heating, *R*_int_ is ∼4% of *R*_w_, showing it has a weak effect on total thermal resistance between the WS_2_ sample and DI water, while under the ns laser this ratio is ∼20%. As a result, we expect to observe the effects of *R*_int_ on the temperature evolution of WS_2_ film under the ns heating state. Also, these two figures represent the thermal diffusion to the water and the fact that *L*_w,ns_ is much shorter than *L*_w,CW_. The red thermal contour in each figure shows this effect. Also, the time-dependent temperature evolution under laser irradiation is represented schematically in the inset of each figure. For the CW case, the temperature rise (Δ*T*_CW_) is constant due to the steady-state heating of this laser. The transient temperature rise and Raman weighted average temperature rise of the ns case are shown using red and orange curves in the inset of part (b). Also, the blue curve indicates a single ns laser pulse.

Two different sizes of holes are made on an Si substrate using FIB to prepare the suspended samples. One of the holes is circular with a diameter of 10 μm and the other one is square with 22 μm side length. Fig. 1 shows the cross-sectional view of the hole that is used to suspend the sample on top of it. Then, three nm-thick WS_2_ flakes are prepared using the mechanical exfoliation method from bulk WS_2_, which guarantees the quality and crystallinity of the layers. Mechanical exfoliation makes it possible to prepare several samples of different thicknesses depending on the force applied to the bulk sample. Finally, these samples are transferred to the holes by gel-films and a 3D micro-stage. More details of this process can be found in our previous work.^45,46^

The Si substrate with the WS_2_ film on top of it is mounted on a stage inside a glass container. This container is filled with DI water. Using this setup, the WS_2_ film is in contact with air from the bottom, while touching the water on top (Fig. 1). Comparing the heat transfer on both sides of the WS_2_ layer, this design guarantees that heat transfers to water as much as possible and maximizes the effect of the water–WS_2_ interface on the temperature evolution of the film. A glass slide is placed on top of the container to prevent water evaporation and to stabilize water inside the container. It should be noted that water will not penetrate underneath the WS_2_ layer in the first few hours during which the Raman experiment is being performed. We observe that after 24 hours or more, a few micro-bubbles are formed beneath the WS_2_ layer, which shows water penetration. As will be mentioned in the next section, the nET-Raman technique is based on the ratio of the temperature rise of the sample under two different heating states; therefore, any constant parameter that contributes equally under both states will have a negligible effect on the measured interface thermal resistance. Placing the glass substrate on top of the container obviously affects the laser power irradiating the sample, but since the transmission of the glass slide under two heating states is the same, it will not affect our measurement and is not considered in the characterization process.

This method can also be applied to other materials, such as bulk ones, by constructing an appropriate geometry. For instance, for bulk silicon with a thickness in the order of 100 s of micrometers, it is possible to drill/cut a hole at a micrometer dimension from the bottom of the Si, in such a way that only a thin layer of Si remains on the top, and its bottom is totally in contact with air. Again, by putting this sample inside a DI water chamber, its top surface will touch the water, and the interfacial thermal conductance between the Si layer and water could be measured.

### 2.2. Physical principles of nET-Raman

The temperature rise of the suspended sample under laser irradiation is directly related to the thermal conductivity of the WS_2_ film (*k*), the thermal conductivity of water, and the interfacial thermal resistance at the water–WS_2_ interface 
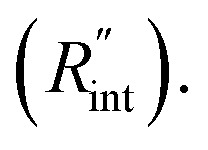
 Temperature changes of the sample could be investigated by studying the frequency variation of Raman-active optical phonons under laser heating. In the nET-Raman technique, two different energy transport states are constructed to analyze the thermal response of the material. Under the first state, the thin sample is irradiated using a continuous-wave (CW) laser to construct steady-state heating. Under this state, the temperature rise of the sample is mainly controlled by the in-plane thermal conductivity of the sample (*k*) and the thermal conductivity of water. The second state, which is a transient state, is a nanosecond (ns) state. This state is constructed using a 300 kHz ns pulsed laser. Under this state, the temperature rise of the film receives more effects from 
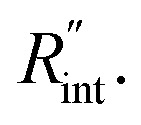


The contribution of 
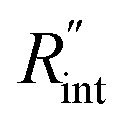
 to the total thermal resistance between WS_2_ and water is more significant in the ns case than in CW. For the CW heating state and under the area of laser heating, the thermal resistance of water *R*_w_ could be estimated as: *R*_w_ = 1/(2*D*_CW_*k*_w_), where *D*_CW_ and *k*_w_ are the laser spot diameter of the CW laser under a 20× objective lens and thermal conductivity of water, respectively. Taking *k*_w_ ≃ 0.6 W m^−1^ K^−1^ for water, and *D*_CW_ = 3.6 μm (Table 2, see below), *R*_w_ will be around 2.3 × 10^5^ K W^−1^. The total interface resistance (*R*_int_) can be estimated as: 
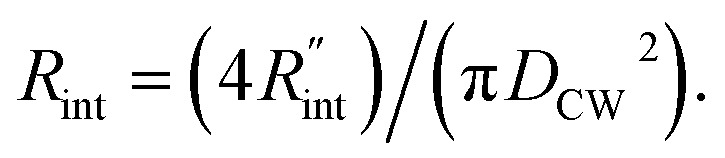
 Take 
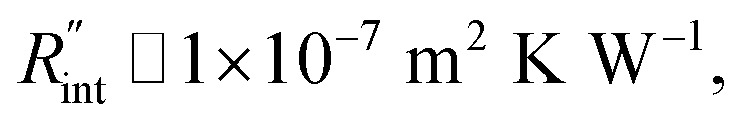
 the total interface resistance will be around 9.8 × 10^3^ K W^−1^, which is 4% of the total water resistance covering the WS_2_ film. Therefore, the interfacial thermal resistance plays a negligible role compared with *R*_w_ in controlling the temperature of the WS_2_ film under the CW state and it is hard to detect its effects under this heating state [Fig. 1(a)]. It should be noted that performing the Raman experiment using a CW laser is necessary in this method, since it leads to the cancelling of the effects of several known and unknown parameters, such as laser absorption and temperature-dependent Raman coefficients, on the final results. This idea is represented in detail in the following paragraphs.

The laser pulse width (*t*_0_) of the ns laser used in this work is 212 ns. During ns laser pulse heating, the thermal diffusion length to the water layer can be estimated as: 
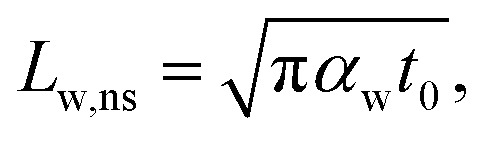
 where α_w_ is the thermal diffusivity of water. *L*_w,ns_ is around 300 nm. The total thermal resistance caused by water under the ns state is estimated as: *R*_w_ = 4*L*_w_/(π*D*_ns_^2^*k*_w_), where *D*_ns_ is the laser spot diameter of the ns laser under a 20× objective lens, which is around 2.5 μm. *R*_w_ under this state is ∼100 × 10^3^ K W^−1^. While this time *R*_int_, using the same estimation as in the CW case and taking *D*_ns_ as 2.5 μm (Table 2), is ∼20.3 × 10^3^ K W^−1^, which is ∼20% of *R*_w_ [Fig. 1(b)]. Hence, we expect that 
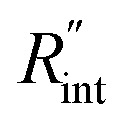
 will play an important role under transient heating in the thermal response of the sample. Fig. 1(a) and (b) show a graphical representation of the relative effects of *R*_w_ and *R*_int_ under both states on total thermal resistance. Also, note that the thermal diffusion length to water under the CW state can be estimated as: *L*_w,CW_ ≃ 10*D*_CW_, which is ∼36 μm. This significant difference between *L*_w,CW_ and *L*_w,ns_ is also schematically shown in these two figures by red thermal contours.

In both states, laser heating and Raman signal excitation take place simultaneously. Collecting this Raman signal under various laser powers could be used to track the temperature evolution of the sample. In fact, we can obtain the Raman shift power coefficient (RSC) under each state by irradiating the sample using several laser powers (*P*). RSC is defined as: *ψ*_CW_ = ∂*w*/∂*P* = *α*(∂*w*/∂*T*)*f*(*k*), where *α* and ∂*w*/∂*T* are the laser absorption coefficient and Raman shift temperature coefficient, respectively. Under an ns energy transport state, which is designed to probe localized heating, RSC can be obtained as: 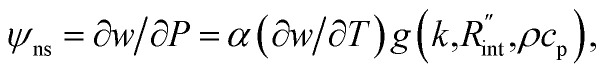 where *ρc*_p_ is the volumetric heat capacity of each WS_2_ thin film. The thermal conductance at the water–WS_2_ interface is defined as:
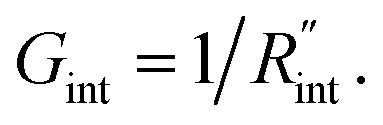
 These definitions of 
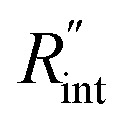
 and *G*_int_ are consistent in the rest of this article. As mentioned earlier, due to the localized heating of the ns state, the contribution of 
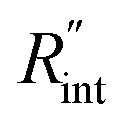
 to *ψ*_CW_ is almost negligible in comparison to *ψ*_ns_; therefore the Raman shift power coefficients are different under these two states. Note that the *f* and *g* functions depend on the thermal properties of the materials under each heating state, and are more complicated to solve analytically. Therefore, it is too complicated to show their analytical forms, and they have to be solved numerically.

Using the last two Raman shift power coefficients *ψ*_CW_ and *ψ*_ns_, a new experimental parameter is defined as: *Θ*_exp_ = *ψ*_ns_/*ψ*_CW_, which is called the normalized Raman shift power coefficient. It can easily be shown that *Θ*_exp_ is only a function of *k*, 
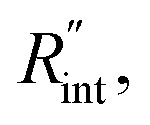
 and *ρc*_p_. And it is no longer a function of the temperature dependent Raman shift coefficient or laser absorption coefficient. This is the beauty of the nET-Raman technique which makes it independent of the last two coefficients. *α* and ∂*w*/∂*T* are generally the main sources of error in steady-state Raman thermometry. Using a 3D numerical model that calculates the temperature rise of the sample under CW (Δ*T*_CW_) and ns (Δ*T*_ns_) heating states, we can find the theoretical value of the temperature rise ratio (*Θ*_th_) as: *Θ*_th_ = Δ*T*_ns_/Δ*T*_CW_. Using known values for *k* and *ρc*_p_ for water and WS_2_, a relationship between *Θ*_th_ and 
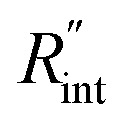
 is found. Finally, this relationship is used to find the 
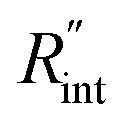
 value that meets the condition: *Θ*_exp_ = *Θ*_th_. As mentioned earlier, known values of *k* and *ρc*_p_ are used here from the literature.^39,47,48^ In the discussion part, it will be shown that both of these values have a negligible effect on the uncertainty and value of measured 
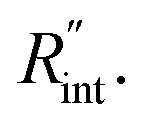


The first part of the 3D heat conduction model deals with steady-state heating under a CW laser, which is governed by the following differential equation:

1 *k∇*^2^*T*_CW_ + *q̇* = 0,

where *T*_CW_ (K) is the temperature in CW heating and *q̇* is the volumetric Gaussian beam heating, which is shown as:2
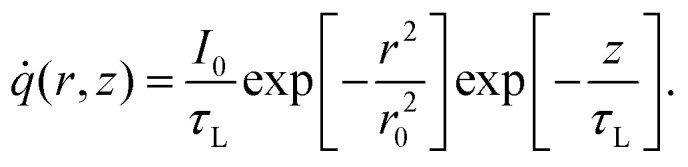
Here *r* is the radial direction that starts at the center of the hole all the way to the boundaries of the suspended area. *z* is the position in the thickness direction. *I*_0_ (=*P*/*πr*_0_^2^) and *τ*_L_ are laser power per unit area at the center of the laser spot and laser absorption depth, respectively. *τ*_L_ is calculated as: *τ*_L_ = *λ*/4π*k*_L_, where *λ* and *k*_L_ are the laser wavelength and extinction coefficient of WS_2_ at corresponding *λ*, respectively. In this work, *λ* is 532 nm, and at this wavelength *k*_L_ takes the value 0.903.^49,50^ Therefore, *τ*_L_ will be ∼46.9 nm. Although this value of *τ*_L_ is used in our calculation, it should be noted that this parameter has a negligible effect on the measured 
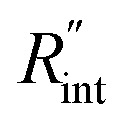
 value using the nET-Raman technique, since it will be canceled out by dividing the temperature rise under two heating states.^48^

Transient-state heating is generated using a 532 nm nanosecond laser with a 212 ns pulse width (*t*_0_). It should be noted that *t*_0_ should be smaller than the time needed for the sample to reach thermal equilibrium (*t*_eq_). This time can be estimated as: *t*_eq_ ∼ (10*r*_0,ns_)^2^/*α*_water_, where *α*_water_ is the thermal diffusivity of water. In this work, *t*_eq_ is around 25 ms, which is much larger than *t*_0_. Another point that is worth mentioning is the effect of hot carrier diffusion on thermal transport in this ns state. In short, as soon as the laser irradiates the WS_2_ sample, electrons in the valence band gain enough energy (more than the Fermi energy) to leave this band, leaving holes behind. These hot carriers recombine within a very short period of time (*t*_l_) which is in the order of 1 ns for WS_2_.^51^ Since *t*_l_ is very much shorter than *t*_0_, we can ignore the effects of hot carrier diffusion on thermal transport. Hot carrier transfer inside TMD materials, such as WS_2_, was well-studied in our previous work.^42,48,52^ Regarding the thermal transport in the cross-plane direction of the WS_2_ sample, it is assumed that the temperature distribution in this direction is uniform. In the thickness direction, heat diffusion length (*L*_⊥_) under ns pulsed laser heating can be estimated as: 
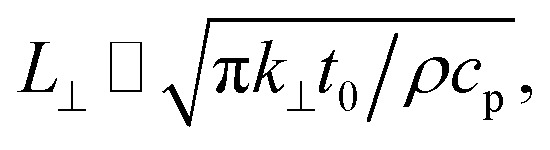
 which is around 1 μm. Here, *k*_⊥_ is the thermal conductivity of WS_2_ in the cross-plane direction and is about 2 W m^−1^ K^−1^.^53^ This value is much larger than the thickness of all samples (Table 1, see below), which confirms the validity of this assumption. The governing equation of the ns laser heating state is:^54^3
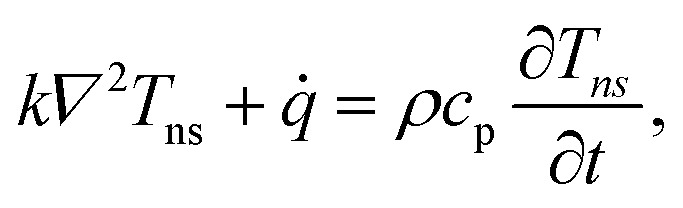
where *T*_ns_ is the temperature under the ns heating state. The heat source term in this state is written as:4
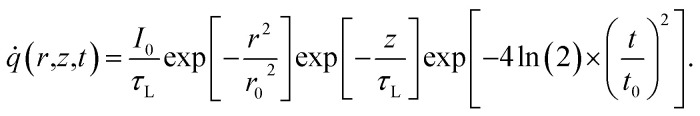
Here, *I*_0_ (W m^−2^) is the peak laser intensity. Additionally, the temperature distribution at the water–WS_2_ interface could be shown as: 
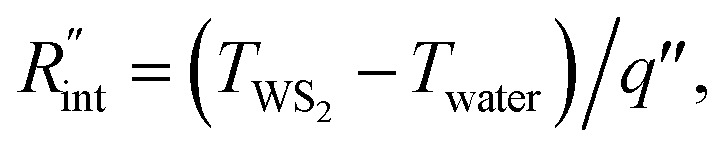
 where *q*′′ is the interface heat flux. Note that *T*_water_ and *T*_WS_2__ are the temperature of the water and WS_2_ film just close to the interface. Using the abovementioned equations, the temperature rise of the sample under two heating states could be calculated for different 
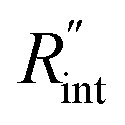
 values. As mentioned earlier, the ratio of these calculated temperature rises of the two states is equal to the experimental normalized RSC for the objective 
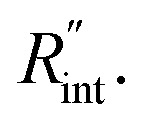
 It worth noting that the experimental RSC is based on Raman intensity-weighted temperature rises in both space and time domains and this point is considered in the theoretical calculation of the temperature rise under each state. Note that the temperature at the edge of the suspended area could be considered to be room temperature under both CW and ns cases for two main reasons. First, the interfacial thermal resistance at the WS_2_–Si interface at the edge is much smaller than the in-plane thermal resistance of the WS_2_ film. Second, the thermal resistance of Si is very low due to its high thermal conductivity. Therefore, it is reasonable to consider the room temperature boundary condition at the WS_2_–Si interface.

**Table 1:** Summary of sample thickness and *R*_q_

Sample	Hole structure	Thickness (*t*) [nm]	Roughness (*R*_q_) [nm]	(*R*_q_/*t*) × 100
1	Square	88	6.20	7.04
2	Circle	33	4.54	13.7
3	Circle	22	2.44	11.1

## 3. Result and discussion

### 3.1. Sample characterization

**Fig. 2:**
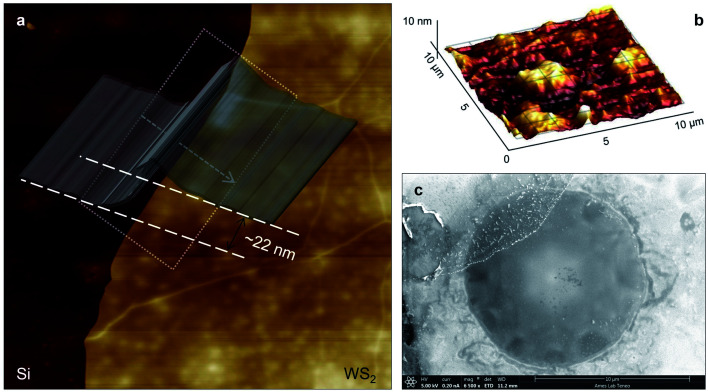
(a) 2D AFM image of Sample 3 at the Si–WS_2_ boundary. The 3D thickness profile represents the average thickness of the sample over the dotted rectangular area. (b) AFM image of a supported area of this sample in the suspended area's neighborhood. The root mean square roughness (*R*_q_) over this area is 2.44 nm. (c) The SEM image of the suspended area shows that this area is smoother and more uniform than the supported area. Also, it shows that the WS_2_ film is not totally flat and is a little bit concave toward the bottom of the trench.

Three suspended samples are prepared using the mechanical exfoliation method. Both AFM and SEM characterizations are performed to study the thickness and roughness profiles, and structure of these films. Fig. 2(a) shows the 2D AFM image of Sample 3 at the boundary of WS_2_ and the Si substrate. AFM measurements are conducted over the supported area to prevent sample damage. The thickness profile of this sample is shown in the figure using a gray 3D thickness profile and corresponds to the average thickness over the dotted rectangle in the direction of the arrow. The thickness of this sample is 22 nm. Fig. 2(b) indicates the 3D AFM image of this sample over a 10 μm × 10 μm area close to the suspended area. The root mean square (RMS) roughness of this sample is measured using this image and is 2.44 nm. Table 1 includes the thickness and roughness values of all samples, as well as the ratio of roughness over thickness. This ratio for all samples is less than 15%, which indicates good contact between the WS_2_ film and the Si substrate.

As will be discussed in the next section, sample roughness is one of the main parameters that can affect 
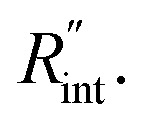
 In order to further study the sample's structure, we performed SEM measurement over the suspended area. Fig. 2(c) shows the SEM image of Sample 3. It shows that the suspended area is almost uniform in all directions. Also, it indicates that the sample is not totally flat over the hole, and is concave toward the bottom of the hole. This will affect the laser spot radius measurement and alter the actual size of the suspended area, and therefore the theoretical temperature rise calculation under both states will vary to some degree. This effect is discussed in detail in the next section.

### 3.2. Water–WS_2_ interface thermal conductance

**Fig. 3:**
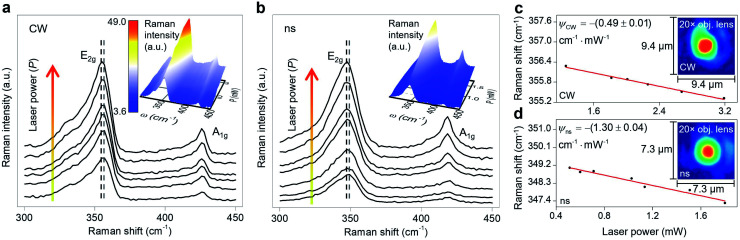
Raman spectra of WS_2_ nm-film (Sample 3) under (a) CW, and (b) ns heating states. Both plots show that the Raman intensity of the E_2g_ and A_1g_ modes increases with increased laser power, and the peak position redshifts with the increased laser power. Here the E_2g_ peak is used to perform the analysis and measure the interfacial thermal resistance 
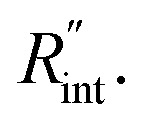
 Two dashed lines in both figures indicate the redshift of the E_2g_ peak. The insets of these two figures represent the 3D contour of Raman intensity as a function of peak position (*ω*) and laser power (*P*). These two contours confirm the aforementioned trends, as well as the linear increase in Raman intensity (*I*) with increased *P*. Note that the I value of the 3D contour of the ns state corresponds to the contour bar that is shown in the inset of part (a). The Raman shift power coefficient (*ψ*) corresponding to the E_2g_ peak of WS_2_ under (c) CW and (d) ns laser of Sample 3. Black dots indicate the experimental position of the E_2g_ peak at different laser powers, and the red line on each plot shows the fitted line to find the *ψ* value under each state. Note that the *x*-axis of both plots is the laser power just after the objective lens and before the laser beam enters the container. Hence, the absorbed laser power under each case is even lower. Since in the nET-Raman technique the ratio of these two RSCs is used to measure 
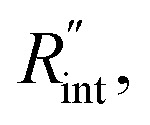
 the laser absorptions of the glass layer, DI water, and WS_2_ sample for each sample are identical for both heating states. This will not affect the determined 
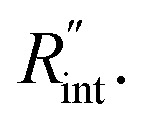
 Ihe inset of each plot shows the laser spots that irradiate Sample 3 under a 20× objective lens for both CW and ns cases.

A room temperature (RT) Raman experiment is conducted using both CW and ns lasers for all three samples to obtain the Raman shift power coefficient. For each sample, based on the WS_2_ film's structure and thickness, optimum laser power is used to find the Raman shift power coefficient with the highest accuracy. For both lasers, a 20× objective lens is used to focus the laser spot onto the surface of the WS_2_ film. This objective is chosen to minimize the effects of hot carrier diffusion on thermal transport. The hot carrier diffusion length (Δ*r*_HC_) is estimated as: 
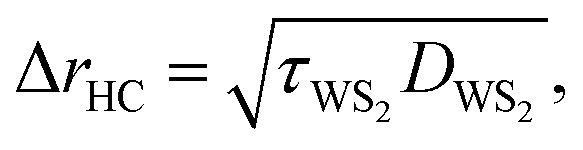
 where *D*_WS_2__ and *τ*_WS_2__ are the hot carrier diffusion coefficient and electron–hole recombination time, respectively. Using *τ*_WS_2__ and *D*_WS_2__ from reference values, Δ*r*_HC_ is ∼0.1 μm, which is much smaller than the laser spot radius under a 20× objective lens.^48^ Therefore, the hot carriers' effects on thermal transport are negligible in our experiment. The radius of the laser spot (*r*_0_) for each Raman experiment is measured by analyzing the optical images of the laser spots based on a Gaussian fitting method. Insets to Fig. 3(c) and (d) show the laser spots of both states for the third sample captured by a CCD camera. As mentioned in the previous section, knowing *r*_0_ for each heating state is necessary to simulate the heating process since it determines the *q̇* in eqn (1) and (3). The laser spot size determines the laser intensity distribution while heating the sample, and, subsequently, the temperature rise and Raman shift. In this work, laser irradiation and laser spot measurement are conducted simultaneously, and the measured *r*_0_ is used directly in our numerical method. Therefore, any effects of laser spot size on our final result are considered precisely. The measured values of *r*_0_ at e^−1^ of the center intensity for all samples are shown in Table 2. Both lasers are operating at 532 nm wavelength. The ns pulsed laser's repetition rate is 300 kHz. For the ns laser, this repetition rate yields ∼4.7 W power at the peak of the laser pulse, and decreasing the repetition rate will increase this peak power and can cause sample damage. As will be shown in the next section, decreasing *t*_0_ without burning the film can reduce the uncertainty level of this technique. More information about the lasers and Raman system can be found in our previous work.^46,55,56^ Also, similar consideration should be involved in choosing the optimum CW laser power to prevent sample damage. Table 2 includes the laser power range for each sample under both heating states.

**Table 2:** Summary of laser spot radii and laser power ranges for three samples under CW and ns states under a 20× objective lens

Sample	CW laser spot radius [μm]	ns laser spot radius [μm]	CW laser power range [mW]	ns laser power range [mW]
1	1.91	1.55	2.21–5.59	0.89–2.27
2	2.17	1.48	1.41–3.55	0.65–1.90
3	1.81	1.32	0.91–3.19	0.51–1.80

As shown in Table 2, the laser spot under the ns laser is smaller than that under the CW laser. This is caused by the collimation difference between the two laser beams. Also, the slight difference between *r*_0_ under each heating state is induced by a variation in focusing level. Note that all of these *r*_0_ values are more than the phonon mean free path (MFP) of the WS_2_ samples (∼15 nm);^51,57,58^ therefore, it is reasonable to assume that thermal transport is diffusive and under local-equilibrium. Additionally, these laser power ranges ensure the linear decrement of Raman shift against increased laser power with minimal local heating effects. Local heating effects induced at higher laser powers can alter the thermal properties of the WS_2_ film and reduce the quality of the experimental data. Note that these laser power ranges are for the laser beam before it reaches the glass cap on top of the substrate. The amount of laser power absorbed by each sample is even less than this and is ∼60%. All of these details are considered in the numerical calculation.

Sample 3 is used here to detail the data processing and the results. Fig. 3(a) and (b) show the Raman spectra of this sample under both heating states by varying the laser power. During the Raman experiment, we did not observe any significant auto-fluorescence in the background while collecting the Raman signal under both lasers. Each spectrum has two main Raman modes: E_2g_ and A_1g_. E_2g_ relates to in-plane vibrations and A_1g_ represents the out-of-plane vibrations. Two dashed lines in this figure indicate the decrease in Raman shift of the E_2g_ mode with increased laser power. The E_2g_ mode is used in this work to find the Raman shift power coefficient, because it is stronger and more suitable for Raman peak fitting. Note that considering the A_1g_ peak and performing the Raman experiment to find the RSC values will not affect the final results. This is shown by conducting nET-Raman using another sample (Sample 4), and the results are reported in ESI.† Ihe insets of these two plots show the 3D Raman intensity contour of this sample under CW and ns states. Also, they indicate that the Raman intensity of both E_2g_ and A_1g_ peaks increases linearly with increased laser power. It can be seen from both contours that both Raman peaks are red-shifted with increased laser power. 2D representations of these two contours are shown in Fig. S2 of ESI.† Note that each point's value in the 3D contour of the ns state follows the contour bar of the inset of Fig. 3(a). All representative Raman spectra of WS_2_, as shown in Fig. 3, are fitted using the Lorentzian function to find the exact Raman shift of the E_2g_ peak at each laser power. The results of this peak fitting are shown in Fig. 3(c) and (d) for CW and ns heating states, respectively. The fitting quality depends on the quality of the experimental data and the Raman peak intensity. Generally, for intensities larger than a certain amount, the fitting quality will be almost intact. In this work, the integration time and laser power are chosen in such a way as to guarantee that the peak fitting uncertainty for low and high power cases are similar and less than 0.02 cm^−1^. Since this value is negligible, it is not included as the uncertainty of measured interface thermal conductance. As mentioned in the previous section, the slope of this line in the low power range indicates the RSC (*ψ*) value as: Δ*ω* = *ψ*Δ*P*, where *ω* is the Raman shift and *P* is the laser power. The *ψ* of the E_2g_ mode under a CW laser is −(0.49 ± 0.01) cm^−1^ mW^−1^, and under the ns laser is −(1.30 ± 0.04) cm^−1^ mW^−1^. Similar results for all samples are included in Table 3. Note that *ψ* under the ns state is generally higher than the steady-state value. This is because for the same average power, the laser peak power of the ns laser is very high and induces a greater temperature rise. Also, the thermal diffusion length under this state is much smaller than the CW value. These two phenomena lead to the higher temperature rise under pulsed laser heating.

**Table 3:** Summary of *ψ* and *Θ*_exp_ values of three suspended WS_2_ films

Sample	Thickness [nm]	*ψ*_CW_ [cm^−1^ mW^−1^]	*ψ*_ns_ [cm^−1^ mW^−1^]	*Θ*_exp_
1	88	−(0.36 ± 0.01)	−(0.80 ± 0.02)	−(2.23 ± 0.09)
2	33	−(0.33 ± 0.01)	−(0.86 ± 0.03)	−(2.63 ± 0.14)
3	22	−(0.49 ± 0.01)	−(1.30 ± 0.04)	−(2.65 ± 0.11)

It can be seen from this table that *ψ* generally increases with decreasing film thickness under each heating state. This is due to the fact that the temperature rise of each sample depends on the amount of absorbed laser energy, *k*, and thickness. The thickness affects the heat conduction in the sample and the laser absorption (multiple reflections in 2D samples and the optical interference effect). Note that for TMD materials, *k* increases gradually with increased thickness for samples of more than ∼5 nm and it reaches the bulk *k* value at larger thicknesses.^45,59,60^

**Fig. 4:**
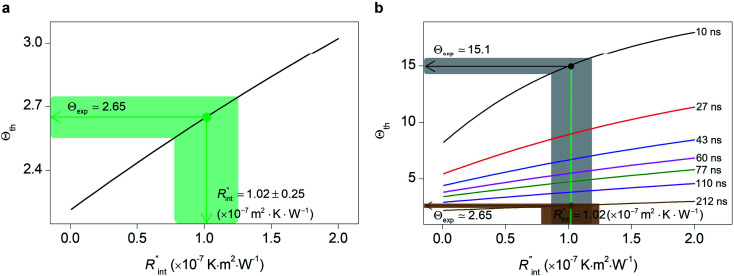
(a) Measured 
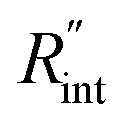
 of Sample 3. The black line represents the theoretical Θ for several 
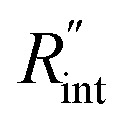
 values, and the green shaded area shows the uncertainty caused by the uncertainty in *Θ*_exp_ on 
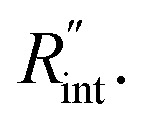
 (b) Investigation of the effects of *t*_0_ on the uncertainty of 
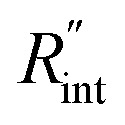
 determined using the nET-Raman technique. This plot shows that 
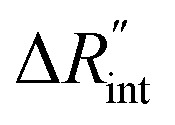
 could be improved by ∼30% when *t*_0_ takes 10 ns compared with the 
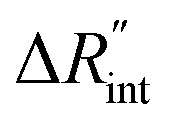
 of the default case where *t*_0_ is 212 ns. This is due to the higher contribution of 
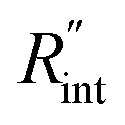
 to the total thermal resistance between WS_2_ and DI water during shorter laser pulse heating.

A 3D numerical calculation based on the finite volume method is conducted to find 
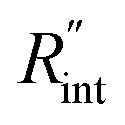
 and consequently interfacial thermal conductance (*G*_int_). The thermal properties of WS_2_ are held constant at: *k* = 32 W m^−1^ K^−1^, and *ρc*_p_ = 1.92 × 10^6^ J m^−3^ K^−1^.^47,48^ Also, the thermal properties of DI water and air are taken from reference values. It will be shown in the following part of this work that uncertainties in these parameters have negligible effects on the determined 
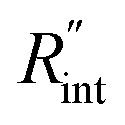
 or *G*_int_ and their uncertainties. Using this simulation, the Raman intensity weighted average temperature rise over both space and time domains for the ns state (Δ*T̄*_ns_), and only over space for the CW state (Δ*T̄*_CW_), are calculated as: 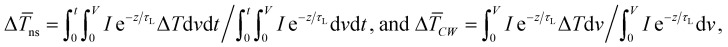 respectively. These two temperature rises are shown schematically in the insets of Fig. 1. The exponential terms (e^−*z*/*τ*_L_^) in these equations are related to the attenuation of the Raman signal as it leaves each scattering location. In these equations, *I*, *V* and Δ*T* represent the laser intensity under each state, sample volume, and temperature rise of each point, respectively. To match the laser intensity with experimental laser heating, the real laser spot radius, as shown in Table 2, is used to perform the simulation. This calculation is conducted for a range of 
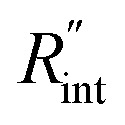
 values and *Θ*_th_ is calculated for each 
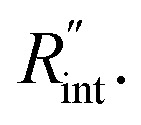
 Finally, the resultant 
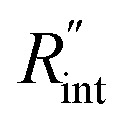
 is deduced by equating *Θ*_th_ to *Θ*_exp_. This process in shown in Fig. 4(a) for Sample 3. Also, the green area represents the uncertainty of the measured 
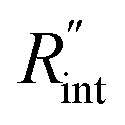
 based on the uncertainty of *Θ*_exp_, as indicated in Table 3. Measured 
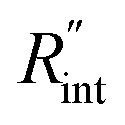
 values, as well as *G*_int_, for all samples are summarized in Table 4.

**Table 4:** Summary of measured 
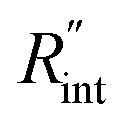
 and *G*_int_ for three suspended samples

Sample	Thickness [nm]	Roughness (*R*_q_) [nm]	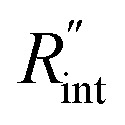 [×10^−7^ m^2^ K W^−1^]	*G*_int_ [MW m^−2^ K^−1^]
1	88	6.20	0.85 ± 0.26	11.8 ± 3.60
2	33	4.54	4.00 ± 0.40	2.50 ± 0.25
3	22	2.44	1.02 ± 0.25	9.80 ± 2.40

It can be seen from this table that the *G*_int_ values of the three samples are almost in the same order, especially for samples 1 and 3. The larger resistance at the WS_2_–water interface of the second sample compared with the other samples could be caused by several factors. First, although the roughness of this sample is in the same order as that of the other two (Table 1), *R*_q_ is over the supported region close to the suspended area and the surface roughness of samples over the suspended region could be different than *R*_q_, especially for the second sample.

### 3.3. Discussion

The measured *G*_int_ values in this work are in good agreement with the reference values of solid–water interface thermal transport measurements. Results from other work as well as the current work are summarized in Table 5.

**Table 5:** Summary of measured *G*_int_ in this work and works conducted by other groups for solid surfaces in contact with water

Solid material	*G*_int_ [MW m^−2^ K^−1^]	Technique (or method)	Ref. #
Si	0.20–333	MD	23
Si	66.7	MD	26
Si	92.8	MD	27
Au	80.4–101	MD	27
Pt	130	Experimental	30
AuPd	100–300	Experimental	31
WS_2_	2.50–11.8	Experimental	This work

Comparing our result with other experimental work, it is obvious that the *G*_int_ of the WS_2_–water interface is an order of magnitude smaller than the *G*_int_ at AuPd–water or Pt–water interfaces, as shown in Table 5. The main factor that could contribute to this is the difference between the surface wettability of these three solids. Generally, Au and Pt possess smaller water contact angles than WS_2_, which means that these surfaces are more hydrophilic than a WS_2_ surface. For clean Au and Pt surfaces, the room temperature contact angle (*θ*_CA_) at atmospheric pressure is in the range of 5–40°.^61–65^ While the *θ*_CA_ of multilayer WS_2_ at RT is around 50–80°.^66,67^ Also, in these works, the solid surfaces are more uniform and are in form of nanoparticles, and are smoother compared with the WS_2_ samples used in our experiment. *θ*_CA_ depends significantly on surface microscale roughness. As discussed in the introduction, hydrophobicity is one of the main parameters that affects the thermal transport at a solid–water interface and a lower *θ*_CA_ leads to stronger solid–water contact. A similar argument is valid regarding the *G*_int_ of ref. 27. Regarding the MD simulation results, it should be noted that ref. 26 reports *G*_int_ at several temperatures from 350 to 550 K, and at temperatures closer to RT, *G*_int_ is of the same order as our results.

As mentioned earlier, one parameter that affects the accuracy of our measurement is ns laser pulse width *t*_0_. As *t*_0_ takes smaller values, the thermal diffusion length in water will be shorter, and 
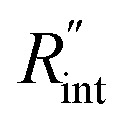
 contributes more to the thermal transport under the ns state compared with longer *t*_0_ cases. To show this fact, the temperature rise of the 22 nm sample under ns is calculated *versus*
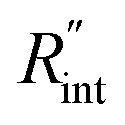
 for several *t*_0_ cases ranging from 10 to 212 ns, and subsequently, *Θ*_th_ is calculated for each *t*_0_ case. Fig. 4(b) shows the result of this calculation. It is reasonable to see that *Θ*_th_ increases with decreased laser pulse width, since shorter pulses means higher pulse peak power that leads to a higher temperature rise. Also, as shown in Fig. 4(b), *Θ*_th_ is plotted for each case. This figure shows that the slope of each 
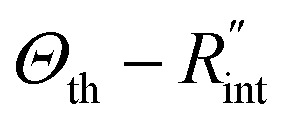
 curve increases with decreased *t*_0_. Now, considering 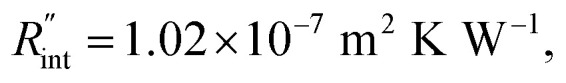 as indicated in Table 4, and assuming constant 5% uncertainty for each hypothetical *Θ*_exp_ value, we can find the uncertainty in 
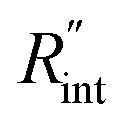
 for each *t*_0_ case. This is shown by the shaded areas in Fig. 4(b) for two extreme cases when *t*_0_ takes 212 ns and 10 ns. It is obvious that this area for smaller *t*_0_ values is narrower that for larger *t*_0_ values, which means higher accuracy in the measurement of 
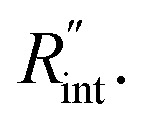
 As mentioned earlier, the 
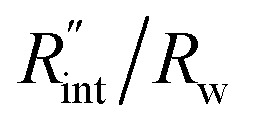
 ratio is ∼20% when *t*_0_ = 212 ns. Similar calculation shows that when *t*_0_ is 10 ns, this ratio is ∼60%, which indicates a higher contribution of interfacial thermal resistance to total thermal resistance between the WS_2_ film and water under ns laser heating. Note that under the ns pulsed laser that is used in this work, when *t*_0_ is 10 ns, the peak power of each laser pulse is ∼12 kW, and could damage the suspended film. Another note worth mentioning is that depending on the increment in laser intensity, the light absorption could be linear or non-linear. As long as the laser intensity is not so high as to make the light absorption non-linear, the laser pulse width could be decreased to increase the sensitivity of *G*_int_ measurement. An alternative way to implement this experiment with smaller pulse widths is using an amplitude modulated frequency laser, with appropriate frequency and narrow pulse. Under such conditions, the pulse width can be short enough to measure 
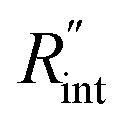
 more accurately, while the laser power is kept below the damage threshold.

**Fig. 5:**
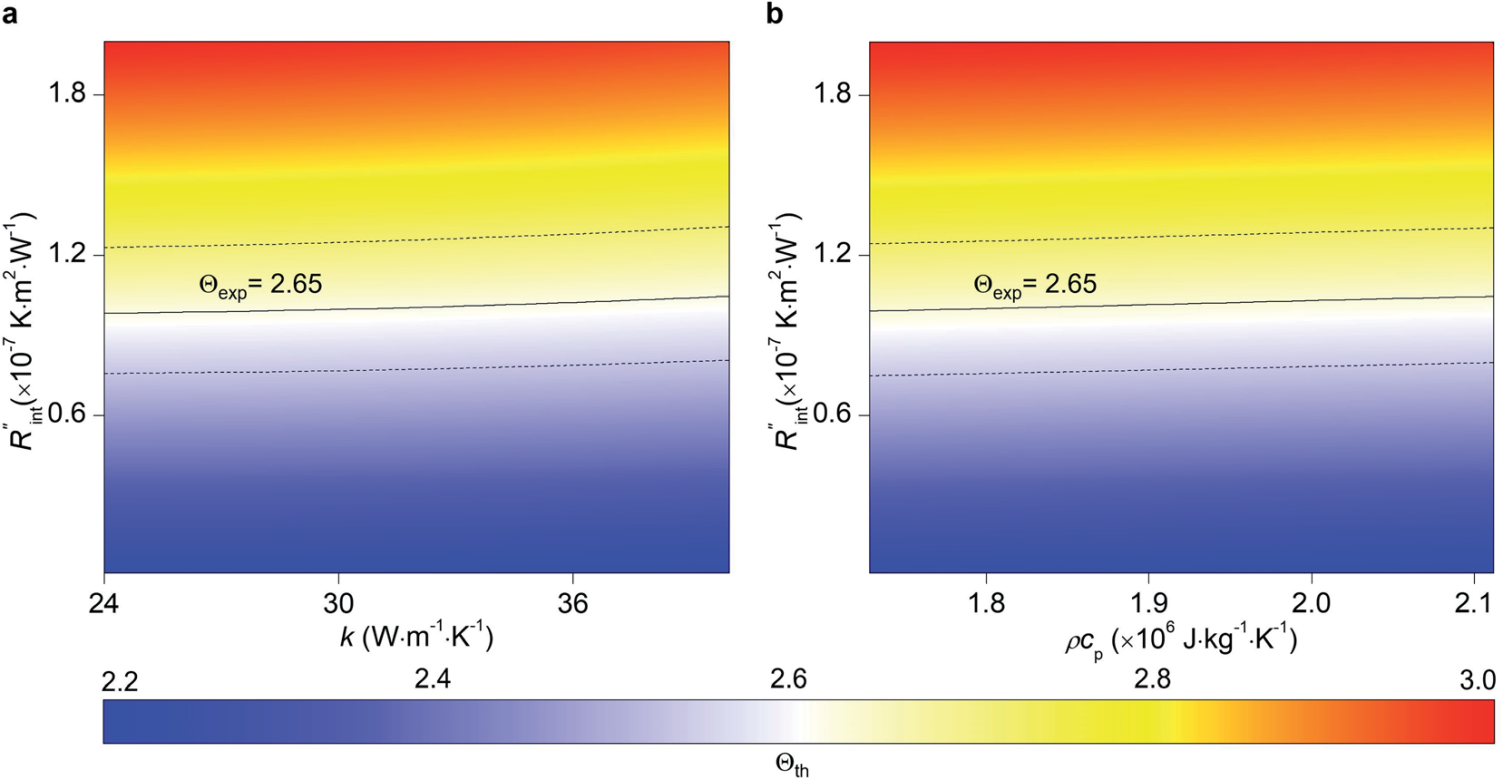
Effects of (a) in-plane thermal conductivity (*k*) and (b) volumetric heat capacity (*ρc*_p_) of WS_2_ thin film on measured 
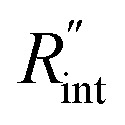
 in this work. Each contour shows the calculated *Θ*_th_ for a range of *k* and *ρc*_p_ of Sample 3, and the solid black line indicates the *Θ*_exp_ of this sample corresponding to Table 3. The two dashed lines on each figure are related to the uncertainty in measured 
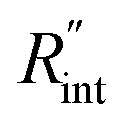
 caused by uncertainty in *Θ*_exp_. Both plots validate the idea that each of these parameters has a negligible effect on the measured 
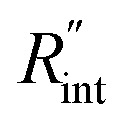
 and 
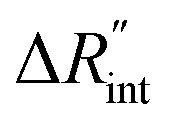
 in the nET-Raman method.

Another study is conducted to show that the nET-Raman technique does not depend on the known values of *k* and *ρc*_p_ of the WS_2_ film. To do so, the temperature rise of Sample 3 under both heating states, and consequently *Θ*_th_, are calculated for a range of *k* and *ρc*_p_. The results of this calculation are shown in Fig. 5. Using the *Θ*_exp_ of this sample (Table 3), 
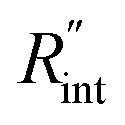
 and its uncertainty are found for each case and represented by black solid line in each plot of Fig. 5. Two dashed lines show the uncertainty of measured 
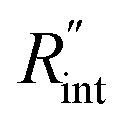
 corresponding to the uncertainty of *Θ*_exp_. These two contours indicate that if *k* and *ρc*_p_ of WS_2_ change by 10% independently, the resulting values of 
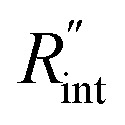
 change by less than 2% and 4%, respectively. Also, the uncertainty of the measured 
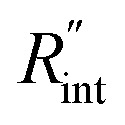
 will be almost intact, since the dashed lines and black solid line in each contour are almost parallel regardless of *k* and *ρc*_p_ values. This figure indicates a critical fact that the effects of *k* and *ρc*_p_ are almost canceled out by introducing Θ in this technique, and the three lines in each contour stay almost horizontal while *k* or *ρc*_p_ is varied.

**Fig. 6:**
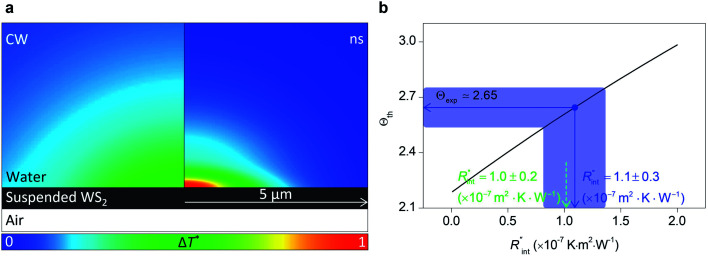
(a) Normalized local temperature rise under CW (left contour) and ns (right contour) cases. These contours show that the local temperature rise at the edge of the suspended area, especially in the ns case, is almost zero. And the area under the laser spot contributes most to the Raman weighted average temperature rise that is used in nET-Raman to find interfacial thermal conductance. (b) Determined 
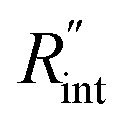
 using the assumption that the suspended sample is not totally flat and is concave inward 1.5 μm toward the bottom of the hole. Under this situation, the heating area domain and *r*_0_ under both states are altered, and updated values are used in the 3D numerical calculation to find 
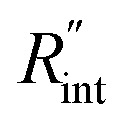
 for Sample 3. The green dashed arrow in this plot shows the measured 
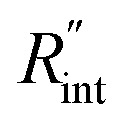
 for flat WS_2_ film, as reported in Table 4. The error caused by this change in the sample diameter on measured 
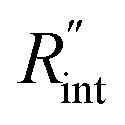
 is less than 8%.

As shown in Fig. 1 and 2(c), we can see that the suspended film is slightly concave toward the hole. In all the aforementioned theoretical calculations that are used to determine 
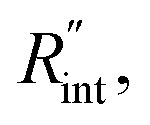
 it is assumed that the suspended sample over the hole is completely flat. To check the uncertainty caused by this assumption, a more realistic case is considered. Here, we assume that the center of the sample is concaved 1.5 μm inward, which is an exaggerated case. The new length of the sample (*l*_arc_) which is the length of the WS_2_ arc over the hole is ∼10.6 μm for a 10 μm hole. This value is used in our theoretical calculation to find the corresponding interface resistance. Fig. 6(b) shows the results of this study. The measured 
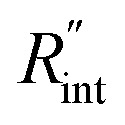
 with similar *Θ*_exp_ for Sample 3 (Table 3) is ∼1.1 × 10^−7^ m^2^ K W^−1^. The uncertainty caused by this elongation in 
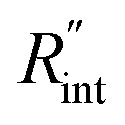
 is ∼7% (Table 4). Therefore, this film elongation has a negligible effect on the determined 
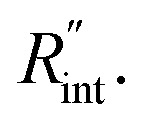


The temperature rise of water at each point in the close vicinity of the WS_2_ film is calculated and plotted in Fig. 6(a), for both CW and ns cases. Here the normalized local temperature rise (ΔT*) is reported. To find ΔT* at each point, the local temperature rise at that point (Δ*T*) is divided by the maximum local temperature rise under ns laser heating (Δ*T*_ns_). This plot shows that ΔT* is mostly increased under the laser spot area, and at higher radii close to the boundaries of the suspended region it is a minimum, and in the case of the ns heating state it is almost zero. This further proves the fact that a minimal increase in the sample length will not affect the Raman weighted average temperature rise of the sample, since the thermal transport mostly occurs under the heating region and not in further away areas.

As mentioned in the main text of the paper, the sensitivity of our technique is mostly controlled by the ns state. The contributions of the thermal resistance of water (*R*_w_) and interfacial resistance (*R*_int_) under this state were elaborated in Section 2.2. The ratio of these two values could be written as: 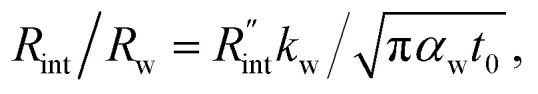 where *k*_w_, *α*_w_, and *t*_0_ are the thermal conductivity and thermal diffusivity of water and laser pulse width. 
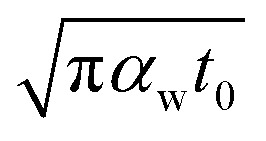
 represents the thermal diffusivity length in water during laser pulse heating. Therefore, the interfacial thermal conductance (*G*_int_) could be written as: 
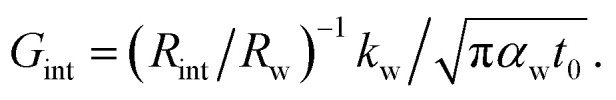
 Assuming that a sensible ratio of these two thermal resistance values is 10% and the laser pulse width is short and is in the order of 5 ns, the upper limit of measurable *G*_int_ would be around 130 MW m^−2^ K^−1^. Also, for similar *t*_0_, the upper limit of measurable *G*_int_ when the sensible *R*_int_/*R*_w_ is 5% will be around 260 MW m^−2^ K^−1^.

## 4. Conclusion

In this work, for the first time, the thermal conductance (*G*_int_) at a liquid–2D material interface was characterized using a novel nET-Raman technique. Two heating states, steady and transient, were introduced to perform the Raman thermometry. Each WS_2_ film was suspended on a micron-sized hole on a Si substrate and this stage was placed inside a glass container filled with DI water. It was reported that *G*_int_ is in the order of ∼10 MW m^−2^ K^−1^ for three measured samples and this was compared and verified with other theoretical and experimental works. It was shown that the surface wettability has a significant effect on the interface thermal conductance. A lower contact angle will lead to significantly increased interface thermal conductance. The nET-Raman technique eliminates the effects of laser absorption coefficient and Raman temperature coefficient on the measured parameters. Also, it was shown that any uncertainty caused by uncertainties in thermal properties from reference values has negligible effects on our characterization. Our rigorous calculation showed that shorter ns laser pulses will significantly increase the effect of interface thermal conductance and improve the measurement uncertainty.

## Conflicts of interest

The authors declare no competing financial interest.

## Supplementary Material

NA-002-D0NA00844C-s001
