# Supplementary material for: Thermal conductance between water and nm-thick WS2: extremely localized probing using nanosecond energy transport state-resolved Raman
Source: Nanoscale Adv. 2020 Nov 2;2(12):5821–32. doi: 10.1039/d0na00844c (PMC9418056; doi:10.1039/d0na00844c)
Supplement: NA-002-D0NA00844C-s001 [file NA-002-D0NA00844C-s001.pdf]

Electronic Supplementary Material (ESI) for Nanoscale Advances.
This journal is © The Royal Society of Chemistry 2020

Supporting Information

Thermal Conductance between Water and nm-thick WS_2: Extremely Localized Probing Using Nanosecond Energy Transport State-Resolved Raman

Hamidreza Zobeiri, [a, †] Nicholas Hunter, [a, †] Ridong Wang, [b, †] Xinman Liu, [c] Hong Tan, [d, *] Shen Xu, [a, e, *] Xinwei Wang [a, *]

[a] Department of Mechanical Engineering, Iowa State University, Ames, Iowa 50011, United States.

[b] State Key Laboratory of Precision Measuring Technology and Instruments, Tianjin University, Tianjin 300072, P. R. China.

[c] Department of Landscape Architecture, University of Washington, Seattle, Washington 98105, United States.

[d] School of Energy and Power Engineering, Nanjing University of Science and Technology, Nanjing 210094, P. R. China.

[e] Automotive Engineering College, Shanghai University of Engineering Science, 333 Longteng Road, Shanghai 201620, People's Republic of China.

[†] H. Zobeiri, N. Hunter, and R. Wang contributed equally to this work.

*E-mails: tanhongwh@njust.edu.cn (H. Tan) , shxu16@sues.edu.cn (S. Xu), xwang3@iastate.edu (X. Wang)

S1. Interfacial thermal conductance (G_{int}) characterization of water-WS_2 nm-film using A_{1g} Raman mode

Another suspended WS_2 film, called Sample 4, is prepared over the 10 μm circular hole, and is used to find R''_{int} and G_{int} by studying the A_{1g} peak of WS_2 sample. Similar sample characterization process is conducted for this sample, and its thickness (t) and roughness (R_q) is measured as 65 nm and 7.09 nm, respectively. Based on this, roughness to thickness ratio ($[R_q/t]\times 100$) is ~11 percent. Also, CW and ns laser spot radii are measured as 2.16 μm and 1.59 μm, respectively. These two values are used in our numerical calculation. Laser power range under CW heating state is 1.11-5.37 mW, and is 0.40-1.93 mW for ns case. Using similar Lorentzian peak fitting, Raman shift of A_{1g} peak for this sample at various laser powers is determined. This is shown in Figure S1(a) & (b) for CW and ns cases, respectively. Using these two figures, RSC values of CW (ψ_{CW}) and ns (ψ_{ns}) modes are found as: -(0.35 ± 0.01) $cm^{-1}\cdot mW^{-1}$ and -(0.67 ± 0.01) $cm^{-1}\cdot mW^{-1}$, respectively. Again, experimental normalized RSC (Θ_{exp}) is calculated as: $\Theta_{exp} = \psi_{ns}/\psi_{CW}$, and is equal to 1.91 ± 0.07. Similar 3D numerical calculation is conducted using this 65 nm sample, and considering the abovementioned laser spot radii, and R''_{int} is determined, as shown in Figure S1(c). Finally, G_{int} is determined to be 10.4 ± 2.65 $MW\cdot m^{-2}\cdot K^{-1}$. This result is in very good agreement with the results that are reported in Table 4 of the paper, and shows that our nET-Raman technique is capable of measuring G_{int} using both E_{2g} and A_{1g} Raman modes.

Additionally, Figure S2 shows the 2D Raman intensity contour of Sample 3 under CW and ns states. It can be seen from both contours that both Raman peaks are red-shifted with increased laser power. Also, these contours indicate that Raman intensity of E_{2g} peak is normally higher than that of A_{1g} peak, which makes fitting E_{2g} peak more reliable and accurate.

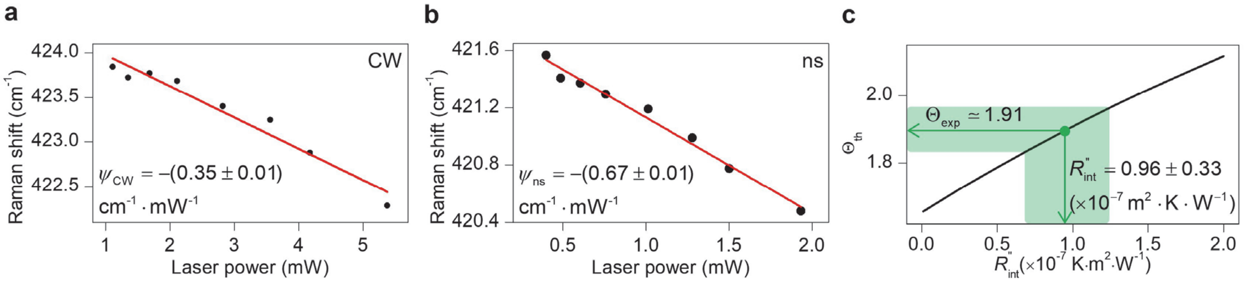

Figure S1. Raman shift power coefficient (ψ) corresponding to A_{1g} peak of WS_2 nm-film under (a) CW and (b) ns laser of Sample 4. (c) Measured R''_{int} and its uncertainty of Sample 4.

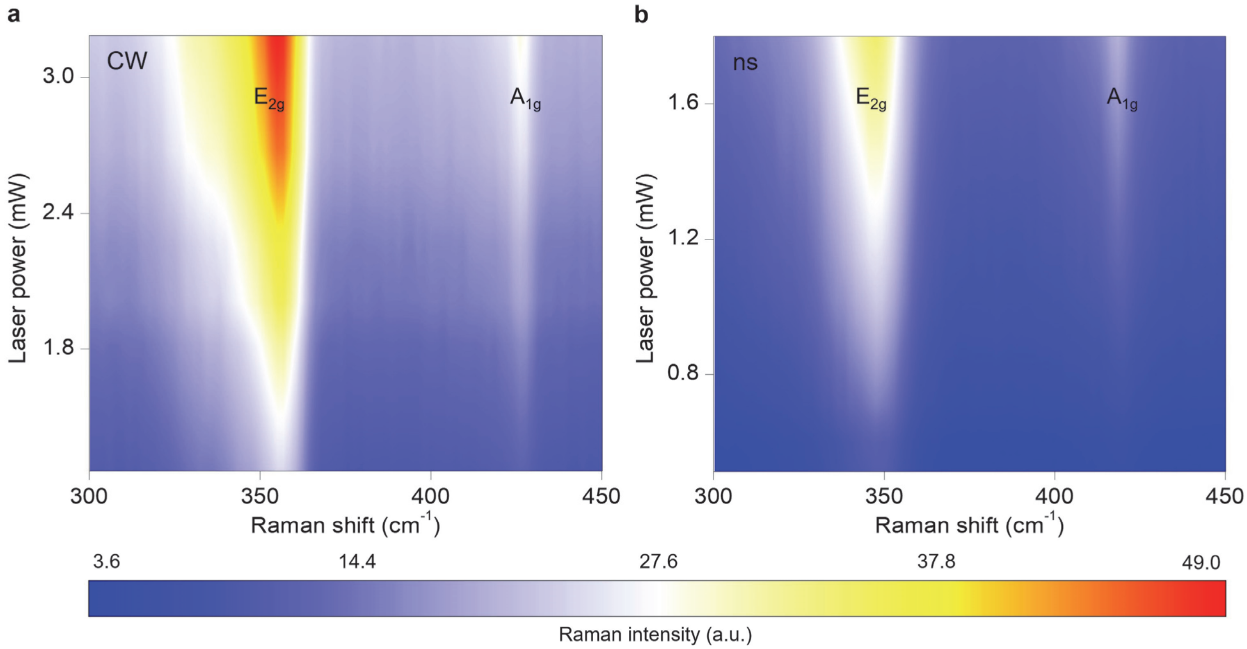

Figure S2. 2D contour of Raman intensity against laser power and Raman shift for (a) CW and (b) ns heating states. These contours show the Raman shift (ω) and Raman intensity (I) variation of E_{2g} and A_{1g} modes of WS_2. Generally, I is higher at higher laser powers (P), and ω redshifts with increased P. Note that these 2D contours are related to Sample 3.
